# Strain and cell type dependent induction of tunnelling nanotube-like projections by African swine fever virus

**DOI:** 10.1186/s13567-026-01866-z

**Published:** 2026-10-30

**Authors:** Brecht Droesbeke, Nadège Balmelle, Hans J. Nauwynck, Herman Favoreel, Marylène Tignon

**Affiliations:** 1 Service Viral Re-Emerging, Enzootic and Bee Diseases, Department Infectious Diseases in Animals, Sciensano, Groeselenbergstraat 99, 1180 Brussels, Belgium; 2 Department of Translational Physiology, Infectiology and Public Health, Faculty of Veterinary Medicine, Ghent University, Salisburylaan 133, 9820 Merelbeke, Belgium

**Keywords:** African swine fever virus, Tunnelling nanotube-like projections, Macrophage subpopulations

## Abstract

African swine fever (ASF) is a devastating viral disease of pigs, causing mortality rates of up to 100%. The causative agent, African swine fever virus (ASFV), preferentially targets cells of the mononuclear phagocytic system (MPS). Despite its severe economic and animal health impact, effective antiviral treatments remain unavailable in Europe. We previously showed that ASFV strain Belgium 2018/01 induces tunnelling nanotube (TNT)-like projections in monocyte-derived macrophages (MDMs), enabling physical connections between distant cells. Here, we investigated the broader relevance of this phenomenon across porcine myeloid cell populations and ASFV strains. We assessed TNT-like projection formation in monocytes, pulmonary alveolar macrophages (PAMs), splenic macrophages, polarized and non-polarized MDMs and Vero cells following ASFV infection. We further compared 20 ASFV strains representing diverse genotypes and virulence levels. Interestingly, luminal monocytes and PAMs did not develop ASFV-induced projections, whereas projection formation increased during monocyte differentiation into MDMs. Splenic macrophages also formed projections, suggesting that interstitial tissue macrophages acquire the ability to induce protrusions. Polarized MDMs exhibited reduced projection formation compared with non-polarized MDMs. Similar structures were induced in Ba71V-infected Vero cells, providing a standardized model for mechanistic studies. Importantly, all 20 ASFV strains induced TNT-like projections in MDMs, although the extent varied substantially and was independent of viral genotype or virulence. Our findings demonstrate that ASFV-induced TNT-like projection formation is a conserved, cell-state-dependent phenotype across diverse ASFV strains. These findings provide a foundation for identifying the cellular and viral determinants of TNT formation and defining its potential contribution to ASFV dissemination and pathogenesis.

**Supplementary information:**

The online version contains supplementary material available at https://doi.org/10.1186/s13567-026-01866-z.

## Introduction

African swine fever (ASF) is an often lethal haemorrhagic disease affecting domestic pigs and wild boar, caused by African swine fever virus (ASFV). Following the virus’s third emergence from Africa into Georgia in 2007, ASF has continued its global expansion, expanding across Europe, Asia, Oceania and America, where it is currently confined to the Caribbean islands [1, 2]. Despite the escalating pandemic threat, no antiviral therapies are currently available in most parts of the world except Vietnam. 

A major obstacle in the development of safe and effective ASF vaccines is the complexity of ASFV itself and its interaction with host cells. ASFV is a large, enveloped, cytoplasmic DNA virus and the sole member of the family *Asfarviridae*. Its genome encodes approximately 170–190 proteins [3], many of which have uncharacterized functions and lack homologues in other viruses or host organisms [3]. ASFV is classified into 23 genotypes (I–XXIII) based on sequence variation within the C-terminal region of the *B646L* gene, encoding the major capsid protein p72 [4, 5].

ASFV predominantly replicates in cells of the mononuclear phagocyte system (MPS), exhibiting a pronounced tropism for tissue-resident macrophages [6]. The capacity of ASFV to replicate within these cells and to induce substantial cytopathic effects in vivo is considered a major driver of viral virulence in domestic swine [7].

Tissue macrophages constitute a key component of the first line of defence and play a central role in linking innate and adaptive immunity, while also contributing to the maintenance of tissue homeostasis. Ontogenically, embryonic precursors transiently present in the yolk sac and the fetal liver give rise to self-renewing tissue macrophage populations [8]. Their functions and phenotypes are highly adaptable and can shift in response to the physiological demands of the surrounding microenvironment [9]. An example of this phenotypical diversity is found in the lungs, where pulmonary alveolar macrophages (PAMs), which reside in the lumen of the alveoli, are phenotypically distinct from the interstitial macrophages, located within the connective tissues, and appear to fulfil different functions [10]. When tissue homeostasis is disrupted by inflammation or infection, circulating monocytes – derived from bone marrow progenitors – are recruited to affected sites via the bloodstream [11]. Following extravasation through diapedesis, these monocytes migrate into peripheral tissues, where they undergo morphological and phenotypic differentiation into monocyte-derived macrophages (MDMs) or dendritic cells under the influence of local growth factors, pro-inflammatory cytokines and microbial stimuli. These newly differentiated cells reinforce or replenish the resident macrophage population [11]. Similarly, during experimental ASFV infection, an increase in circulating blood monocytes has been reported during the acute phase, followed by an accumulation of monocyte-macrophages in infected tissues [12–14].

Within tissues, MDMs display substantial phenotypic and functional plasticity, adjusting their physiological state according to environmental cues [15]. They can polarize toward pro-inflammatory (MDM1) or anti-inflammatory (MDM2) phenotypes, which represent the extremes of a continuum encompassing multiple intermediate activation states [16, 17]. These polarization states are associated with distinct transcriptional, metabolic and functional profiles [16]. In vitro, porcine peripheral blood monocytes can be differentiated into non-polarized MDMs after 4 days of culture in the presence of human macrophage colony-stimulating factor (hM-CSF) [18]. Subsequent exposure to lipopolysaccharide (LPS) and interferon-γ (IFN-γ) induces a pro-inflammatory M1-like polarization (MDM1), whereas interleukin-4 (IL-4) drives an anti-inflammatory M2-like polarization (MDM2) [18, 19].

A recent study compared the replication dynamics of a virulent ASFV strain in monocytes, unpolarized monocyte-derived macrophages (MDMs) and two polarized MDM subsets (MDM1 and MDM2) [19]. The virus exhibited a higher replication tropism in MDMs than in either MDM1 or MDM2, both of which were nonetheless more permissive than monocytes, as reflected by the proportion of p72-positive cells and extracellular viral copies 18 h post-infection (hpi). Franzoni et al. speculated that the tested virulent strain likely replicates more efficiently in unpolarized MDMs than in polarized MDM1 or MDM2 [19].

Further, despite the enhanced propensity of ASFV to replicate in tissue macrophages, we previously demonstrated substantial variation in ASFV replication kinetics between MDMs and other primary macrophage populations originating from different tissues, including PAMs and splenic macrophages [20]. Infected PAMs displayed a delayed cell-to-cell spread compared with MDMs and splenic macrophages, manifested by a lower number of infected cells through 48 hpi, the endpoint of the study. Together with the results of Franzoni et al. [19], these observations support the existence of nuanced differences in virus–host interactions across monocyte/macrophage sub-populations, likely influenced by their tissue-specific micro-environments and polarization states during homeostatic conditions. Collectively, these findings highlight MDMs as a physiologically relevant model for investigating ASFV–host interactions, despite their distinct ontogeny, lack of the self-renewing capacity characteristic of bona fide tissue-resident macrophages and inability to fully recapitulate the tissue-specific micro-environmental cues that shape macrophage phenotype and functionality in vivo [14, 19, 21, 22].

Intriguingly, we recently observed the induction of long membrane projections in MDMs infected in vitro with ASFV strain Belgium 2018/01 (BE18) [23]. These structures were capable of establishing contact with distant cells and displayed morphological and functional characteristics consistent with tunnelling nanotubes (TNTs) [24].

Tunnelling nanotubes (TNTs) constitute a mechanism of physiological intercellular communication observed under homeostatic conditions as well as in various pathological contexts, including neurodegenerative disorders, cancer and infectious diseases, most prominently during viral infections [25]. TNTs are distinguished from other cellular protrusions by their thin, straight membrane-tethered appearance, which are consistently supported by filamentous actin (F-actin) as the primary cytoskeletal backbone. Moreover, they extend above the substrate and establish direct physical connections between distant cellular compartments, often spanning multiple cell bodies in length [24]. These membrane protrusions provide a direct conduit for the intercellular transfer of diverse cargo, including ions, proteins, viral particles or genomes and even organelles, enabling communication between spatially separated cells that were initially not in direct contact [26]. In the context of other porcine viral infections, such as porcine reproductive and respiratory syndrome virus (PRRSV) and pseudorabies virus (PRV), similar TNT-like structures have been shown to facilitate cell-to-cell viral dissemination, even in the presence of neutralizing antibodies [27, 28]. However, because TNTs lack definitive molecular markers and the ASFV-induced protrusions exhibited context-dependent features, we designated these structures as TNT-like projections [23].

Overall, previous findings, including those reported by Franzoni et al. [19, 20], indicate that interactions between ASFV and host cells vary among distinct cellular subpopulations of the MPS. However, data regarding the capacity of these different macrophage subsets to generate TNT-like projections following ASFV infection are currently lacking. To address this gap, we systematically compared multiple macrophage subpopulations to evaluate their ability to form membrane protrusions in response to ASFV infection.

Furthermore, the initial study describing ASFV-induced TNT-like projections was limited to a single viral isolate, leaving it unclear whether this phenotype is conserved across diverse ASFV strains. To investigate this, we screened a panel of 20 ASFV isolates, representing 14 different genotypes, including the Vero cell-adapted strain Ba71V (genotype I), for their capacity to induce protrusion formation.

Collectively, these findings provide a comprehensive foundation for future studies aimed at elucidating the biogenetic mechanisms and functional relevance of ASFV-induced TNT-like projections in the context of African swine fever pathogenesis.

## Materials and methods

### Viruses and cells

#### Primary cell preparation

The isolation of monocytes and their differentiation into MDMs was performed as previously described [23]. Briefly, peripheral blood mononuclear cells (PBMCs) were isolated from heparinized venous blood by Ficoll-Paque^TM^ density gradient centrifugation. PBMCs were seeded in T-175 flasks, and non-adherent cells were removed after 1.5 h at 37 °C and 5% CO_2_ by washing 3 times with Hanks’ balanced salt solution (HBSS) (Gibco). Adherent monocytes were cultured overnight in monocyte medium consisting of Roswell Park Memorial Institute (RPMI) medium + GlutaMAX (Thermo Fisher), supplemented with 1% penicillin (Kela), 1% gentamicin (Gibco), 1% amphotericin B (Gibco) and 10% fetal calf serum (FCS; Gibco). Cells were subsequently detached as previously described [29] and cryopreserved at 1 × 10^7^ cells/mL.

Before use, cryopreserved monocytes were thawed and resuspended in  monocyte-derived macrophage medium  (MDM medium), consisting of monocyte medium, supplemented with 50 ng/mL recombinant human macrophage colony-stimulating factor (hM-CSF; Pepro Tech). Cells were allowed to differentiate for 2, 24, 48, 72 or 96 h after seeding before inoculation as described in [18].

MDMs were polarized toward MDM1 or MDM2 phenotypes according to the protocol outlined in [18]. Briefly, after culturing the monocytes for 96 h in MDM medium, the culture medium was replaced by monocyte medium, supplemented with either 10 ng/mL LPS (Sigma), and 100 ng/mL recombinant porcine IFN-γ (Bio-Rad) was added to induce classical (MDM1) macrophage activation, or 10 ng/mL recombinant porcine IL-4 (R&D Systems), to promote alternative (MDM2) macrophage activation.

PAMs and splenic macrophages were isolated as described in [20]. PAMs were cryopreserved in RPMI + GlutaMAX supplemented with 10% DMSO until use. After thawing, PAMs were resuspended at 1 × 10^6^ cells/mL in PAM seeding medium (monocyte medium supplemented with 1% non-essential amino acids and 1% sodium pyruvate) and cultured for 24 h before inoculation.

Splenic macrophages were isolated from mechanically dissociated spleen tissue and further enriched by Ficoll-Paque^TM^ density gradient centrifugation [20]. The subsequent adherence, culture and seeding procedures were identical to those used for MDMs, except that PAM seeding medium was used throughout. Splenic macrophages were cultured for 48 h before inoculation.

All primary cell populations were seeded at 5 × 10^5^ cells/well in 48-well plates (Greiner) containing 8-mm no. 1 glass coverslips (Epredia) to ensure comparable culture conditions. For splenic macrophages, coverslips were pre-coated with 0.01% poly-l-lysine (Sigma), according to the manufacturer’s instructions before cell seeding.

Vero cells (ATCC) were maintained in T-75 flasks (Thermo Fisher) in medium consisting of Dulbecco’s modified Eagle medium (DMEM; Gibco) supplemented with 1% penicillin, 1% gentamicin, 1% amphotericin B and 10% FCS. Confluent monolayers were detached by incubation with 0.25% trypsin–EDTA (Thermo Fisher) for 10 min at 37 °C and reseeded in Vero medium at 5 × 10^4^ cells/well in 48-well plates containing glass coverslips, resulting in approximately 75% confluence after 24 h.

#### Viruses and infection

In this study, 20 ASFV strains were used, including 19 field isolates and one cell culture-adapted strain, representing different genotypes and virulence profiles. Detailed information on all strains is provided in Table 1. The Belgium 2018/01 (BE18) strain was isolated from the spleen of a wild boar found dead in Etalle, Belgium, in 2018 [30, 31]. A selection of ASFV strains representative of the virus’ genotypic diversity and reported differences in virulence was kindly provided by Dr Carmina Gallardo [European Union Reference Laboratory for African Swine Fever (EURL-ASF), Centro de Investigación en Sanidad Animal-Instituto Nacional de Investigación y Tecnología Agraria y Alimentaria (CISA-INIA); Madrid, Spain] and Dr Carrie Batten [World Organisation for Animal Health (WOAH) reference laboratory for ASF, Pirbright Institute; Pirbright, UK] from their respective repository collections.

**Table 1:** **Information on the strains used in the study**

Strain	Origin	Genotype	Virulence	Passage level	Stock titre [log_10_ tissue culture infectious dose (TCID)_50_/mL]	References	
E70	Spain, 1970	I	Virulent	4	6.6		[32]
NH/P68 (NHV)	Portugal, 1968	I	Low virulent	4	6.3		[33]
OURT 88/3	Portugal, 1988	I	Low virulent	2	6.0		[34]
L60	Portugal, 1960	I	Virulent	2	6.5		[35]
Ba71V	Spain, 1971	I	Non-infective	2	7.0		[36]
Belgium 2018/01 (BE18)	Belgium, 2018	II	Virulent	8	6.5		[30, 31]
Est15/WB-Valga-6 (EST)	Estonia, 2015	II	Moderately virulent	5	6.5		[37]
SPEC 57	South Africa, 1985	III	Unknown	2	5.7		[38]
RSA-W1-1999	South Africa, 1999	IV	Unknown	2	6.0		[39]
MOZ/79	Mozambique, 1979	V	Unknown	2	5.3		[40]
MOZ 94/1	Mozambique, 1994	VI	Unknown	2	5.6		[39]
RSA 85/1	South Africa, 1985	VII	Virulent	2	6.1		[41]
Ken 06.Bus	Kenya, 2006	IX	Virulent	2	6.3		[42, 43]
Ken 05/TK1	Kenya, 2005	X	Moderately virulent	2	6.0		[44]
Kab 6/2	Zambia, 1983	XI	Virulent	2	7.0		[45, 46]
MFUE 6/1	Zambia, 1982	XI	Unknown	2	6.7		[45]
SUM 14/11	Zambia, 1983	XIII	Virulent	2	6.0		[45, 46]
NYA 1/2	Zambia, 1986	XIV	Unknown	2	5.7		[45]
Zim 92/1	Zimbabwe, 1992	XVII	Unknown	2	5.7		[47]
RSA 1/92	South Africa, 1992	XIX	Unknown	2	6.0		[48]

With the exception of the Vero cell-adapted strain Ba71V, all virus stocks were propagated on MDMs derived from the same donor animal. Cultures were harvested once a marked cytopathic effect was observed in the majority of cells, after which cells were lysed by three freeze–thaw cycles. Viral supernatants were clarified by centrifugation at 2000 × *g* for 10 min and titrated on PAMs using an immunoperoxidase monolayer assay as previously described [20]. The titres of the virus stocks ranged from 5.3 to 7.0 log_10_ median tissue culture infectious doses per milliliter (TCID_50_/mL) (Table 1).

The Vero cell-adapted strain Ba71V was propagated on Vero cell monolayers in T-75 cell culture flasks and titrated on Vero cells according to the procedure in 20, with the modification that infected monolayers were fixed in 100% methanol at −20 °C for 20 min. TCID_50_ values were calculated using the Reed and Muench method [49].

Virus infections of primary cells were performed as described in 23, and culture medium was used for mock infection. For the comparative analyses of the primary macrophages derived from blood, lungs and spleen, cells were infected with BE18 at a multiplicity of infection (MOI) of 1. For screening of the ASFV strain panel, a MOI of 0.1 was selected, as a MOI of 1 did not permit comparable infection conditions owing to differences in virus stock titres. After infection, the inoculum was removed and replaced with an equal volume of fresh culture medium in all wells (0.5 mL). Vero cells were infected at a MOI of 10 following the same procedure as for primary cells and subsequently maintained in Vero medium supplemented with 2% FCS until fixation.

### Fluorescence microscopy

At the indicated time points, cells were fixed, permeabilized and indirectly immunolabelled as previously described [23]. Detailed information on all primary and secondary antibodies, as well as the chemicals used in this study, is provided in Table 2.

**Table 2:** **Reagents used for fluorescence microscopy**

Target primary antibodies	Clone	Isotype	Working dilution	Supplier
CD172a	BL1H7	Mouse IgG1	1/50	Bio-Rad
CD163	2A10	Mouse IgG1	1/100	Bio-Rad
MHC-II	MSA3	Mouse IgG2a	1/200	Kingfisher Biotech
CD14	MIL2	Mouse IgG2b	1/100	[50]
ASFV major capsid protein p72	1BC11	Mouse IgG1	1/100	Ingenasa

**Secondary antibodies (conjugate)**	**Host/species reactivity**	**Anti-isotype**	**Working dilution**	**Supplier**
FITC	Goat anti-mouse	Anti-IgG1	1/200	Abcam
Alexa Fluor 594	Goat anti-mouse	Anti-IgG2a	1/200	Invitrogen
Alexa Fluor 594	Goat anti-mouse	Anti-IgG2b	1/200	Invitrogen

**Conjugate**	**Cellular target**	**Reagent**	**Working dilution**	**Supplier**
iFluor 594	F-actin	Phalloidin	1/500	Abcam
Unconjugated	DNA	Hoechst 33342	1/1000	Invitrogen

Fluorescence microscopy was used to characterize the different cell populations, determine the percentage of infected cells and quantify the proportion of mock- and ASFV-infected cells displaying TNT-like projections. The operator was not blinded to the sample identity and had access to the sample identifiers during the random selection of fields and subsequent image analysis.

Prior to infection, the proportion of porcine monocytes and macrophages in the isolated primary cell populations was assessed by fluorescence microscopy as previously described [20]. Briefly, PAMs and MDMs were immunolabelled with the porcine macrophage marker CD163, whereas progressively differentiating monocytes were double-labelled with CD14 and CD163 [51]. Owing to the heterogeneous expression of CD163 in splenic macrophages [51], the pan-myeloid marker CD172a was used to identify splenic monocytes and macrophages. After immunolabelling with the appropriate cell-specific markers, nuclei were counterstained with Hoechst 33342. The percentage of marker-positive cells was calculated as the number of positive cells divided by the total number of Hoechst 33,342-positive nuclei in five randomly selected fields per replicate.

The mean fluorescence intensity signal (MFI) of each cell marker was determined by analysing 180 randomly selected cells per condition following confocal image acquisition using a Zeiss Axio Imager 2 confocal laser scanning microscope equipped with a 63 × oil immersion objective [23]. Images were analysed with Nikon Imaging Software (NIS) Elements Advanced Research (Ar) software (Nikon). Cell boundaries were manually delineated by tracing the cell perimeter to define the region of interest for fluorescence intensity measurements.

The percentage of infected cells was determined after immunofluorescence staining of the ASFV p72 antigen followed by nuclear counterstaining with Hoechst 33342. For each replicate, infected cells were quantified in five randomly selected microscopic fields.

The proportion of cells displaying TNT-like projections was determined after immunofluorescence staining of the p72 antigen, F-actin staining with Phalloidin iFluor 594 and nuclear counterstaining with Hoechst 33,342, as previously described [23]. A total of 600 cells was analysed per condition by direct microscopic examination across multiple low-magnification fields. For infected cultures, 600 randomly selected p72-positive cells were evaluated, whereas in mock-infected cultures, 600 randomly selected cells were analysed. Cells exhibiting straight F-actin-positive protrusions extending beyond the length of a single cell body were scored as positive for TNT-like projections. When a straight actin-based protrusion connected two spatially separated nucleated cells, only one of the connected cells was counted as positive.

### Real-time qPCR

DNA was extracted using an automated magnetic bead-based extraction system consisting of the Indimag 48 instrument (Indical Bioscience) and the Indimag Pathogen Extraction Kit, following the manufacturer’s instructions. Subsequently, a duplex real-time quantitative polymerase chain reaction (qPCR) assay targeting the viral p72 gene and the cellular β-actin gene, used as an internal reference, was performed. Amplification was carried out on a LightCycler 480 thermocycler (Roche) according to the protocol previously described in 52.

### Statistics

All data are presented as the mean ± standard deviation (SD) of three independent biological replicates. For each biological replicate, all experimental conditions were analysed in technical triplicates. Statistical analyses were performed using GraphPad Prism 9 (San Diego, CA, USA). Normality was confirmed by Shapiro–Wilk test prior to further analyses. Comparisons among more than two groups were conducted using one-way analysis of variance (ANOVA) followed by Tukey’s post hoc multiple comparison test. Comparisons between two groups were performed using a one-tailed paired Student’s *t*-test. A *p*-value < 0.05 was considered statistically significant.

## Results

### ASFV infection triggers the formation of TNT-like projections in MDMs and splenic macrophages, but not in PAMs

To investigate the impact of macrophage origin on the induction of TNT-like projections following ASFV infection, primary macrophages were isolated from lungs (PAMs) and spleens or were differentiated in vitro from peripheral blood monocytes into MDMs.

**Figure 1:**
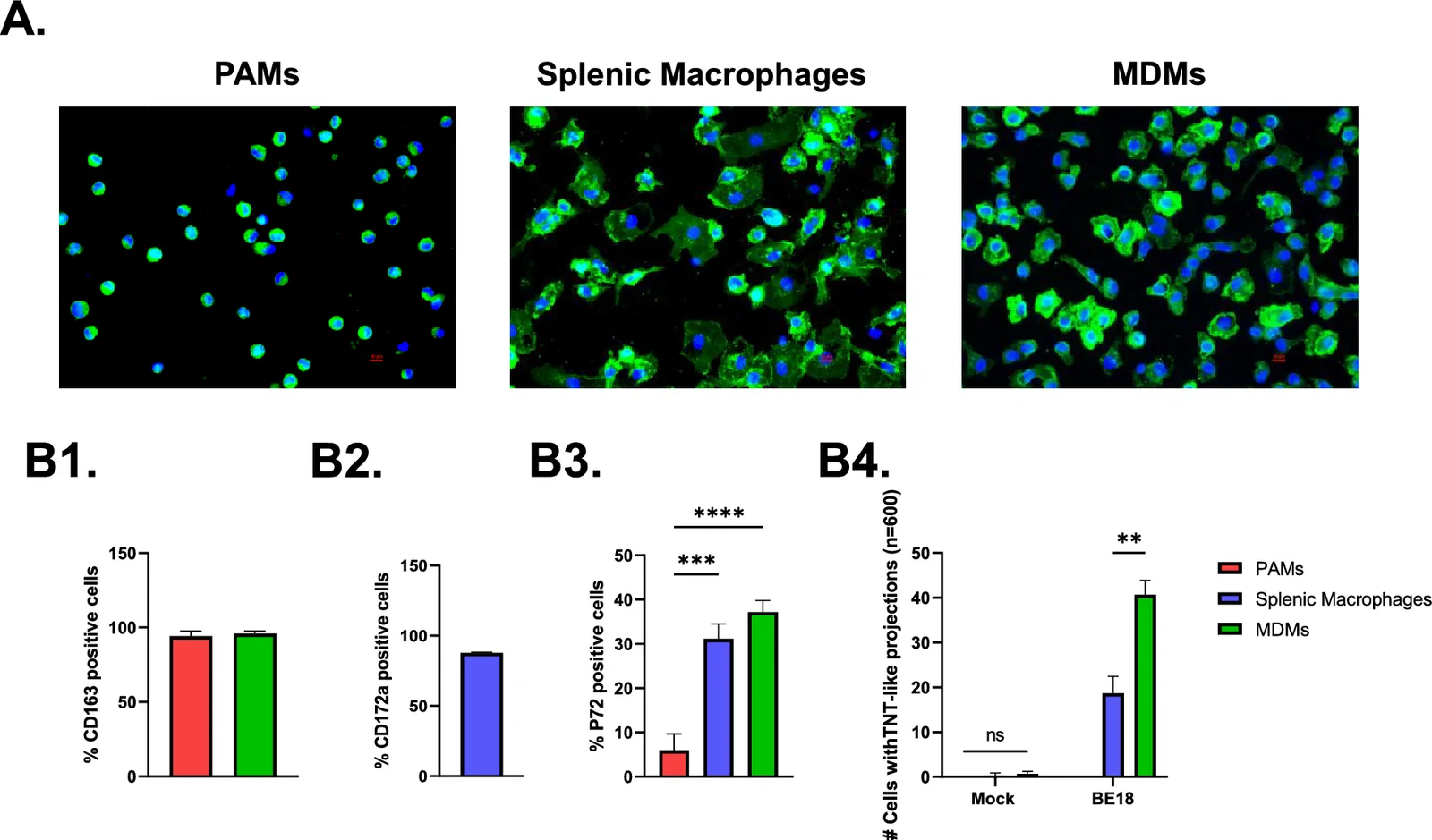
**Comparison of ASFV-susceptibility and TNT-induction in primary macrophages from different tissue origins.** **A** The morphology of porcine pulmonary alveolar macrophages (PAMs), splenic macrophages and monocyte-derived macrophages (MDMs) was assessed by fluorescence microscopy. Cells were stained for macrophage surface markers CD163 (green; PAMs and MDMs) or CD172a (green; splenic macrophages), and nuclei were counterstained with Hoechst 33342 (blue). Scale bar: 10 µm. **B1–B2** The proportion of CD163-positive cells in the isolated PAM and MDM populations **B1** and CD172a-positive cells in isolated splenic macrophage populations **B2** was assessed by immunofluorescence microscopy. **B3** At 18 hpi (ASFV BE18, MOI = 1), cells were fixed and fluorescently labelled to quantify p72-positive cells following visualization of nuclei, the actin cytoskeleton and the viral p72 antigen. **B4** On the same coverslips, 600 mock-infected and 600 p72-positive cells were manually scored for the presence of one or more straight, F-actin-containing cellular protrusions to enable a comparative analysis. Statistical significance is indicated as ^****^p < 0.0001, ^***^p < 0.001 and ^**^p < 0.01.

The fraction of marker-positive cells within the isolated adherent primary macrophage populations was assessed prior to infection by immunofluorescence microscopy. CD163-positive cells were quantified in PAMs (after 24 h of culture) and MDMs (after 96 h of differentiation in MDM medium), whereas CD172a-positive cells were quantified in splenic macrophage cultures 48 h after seeding (Figure 1A, B1, B2).

CD163-positive cells represented the majority of the isolated PAM population (94.13% ± 3.61%). These cells generally displayed a relatively small, spherical morphology and were relatively homogeneous in size and shape. In contrast, CD172a-positive cells accounted for 87.64% (± 0.52%) of the isolated splenic macrophage population and exhibited marked morphological heterogeneity. Most splenic macrophages displayed a spread-out morphology with pronounced variation in cell size and shape, whereas a smaller subset exhibited a rounded morphology resembling that of immature MDMs.

Similarly, CD163-positive cells represented 95.94% (± 1.67) of the isolated MDM population. MDMs exhibited an intermediate morphology between PAMs and splenic macrophages and typically displayed a characteristic “fried egg” appearance. Unlike splenic macrophages, MDMs showed a relatively homogeneous morphology throughout the population.

Overall, splenic macrophages exhibited the greatest morphological heterogeneity and spread morphology, MDMs showed intermediate morphological diversity, whereas PAMs were the most uniform in both cell size and shape.

In addition to this morphological heterogeneity, the three macrophage populations exhibited distinct susceptibilities to ASFV infection (Figure 1B3). At 18 hpi, PAMs showed a relatively low infection rate, with 5.89% (± 3.69) of cells infected. In contrast, splenic macrophages displayed a significantly higher proportion of infected cells (31.15% ± 3.38, *p* < 0.001). MDMs exhibited the highest susceptibility to ASFV infection, with 37.20% (± 2.63) of cells infected at 18 hpi. This infection rate was significantly higher than that observed in PAMs (*p* < 0.0001), in agreement with previously obtained results [20], but did not differ significantly from that observed in splenic macrophages.

Differences between the three cell populations were also observed in the number of ASFV-infected cells exhibiting TNT-like projections (Figure 1B4). Interestingly, ASFV-infected PAMs did not form TNT-like projections. In contrast, 18.67 (± 3.77) out of 600 p72-positive splenic macrophages displayed TNT-like projections. The p72-positive MDMs showed a significantly higher proportion of infected cells with projections (42 cells ± 4.58 out of 600 p72-positive cells; *p* < 0.01) compared with infected splenic macrophages. These results are in contrast with the mock-infected macrophage populations. Mock-infected PAMs, incubated with culture media, similar to infected PAMs, displayed no cells with protrusions, whereas mock-infected MDMs and spleen macrophages presented relative low numbers of cells with TNTs, namely 0.67 (± 0.58) and 0.33 (± 0.58) out of 600 cells, respectively.

Overall, MDMs showed the highest susceptibility to ASFV infection, followed by splenic macrophages and PAMs. Similarly, MDMs exhibited the highest frequency of BE18-induced TNT-like projections, with splenic macrophages showing an intermediate number of cells with projections. This is in contrast to PAMs, where infection did not result in protrusion formation.

## The quantity of TNT-like projections increases with maturation time of monocytes towards MDMs

To verify and characterize the stepwise differentiation of adherent monocytes into monocyte-derived macrophages (MDMs), monocytes were cultured and analysed at 24 h intervals from the onset of cultivation. At each time point from 2 h, and up to 72 h of cultivation after seeding, expression levels of the monocyte marker CD14 and the macrophage marker CD163, together with changes in cell surface area, were assessed to monitor progressive maturation. CD163 was additionally used to evaluate the proportion of monocyte/macrophages in the isolated populations during the different stages of maturation.

**Figure 2:**
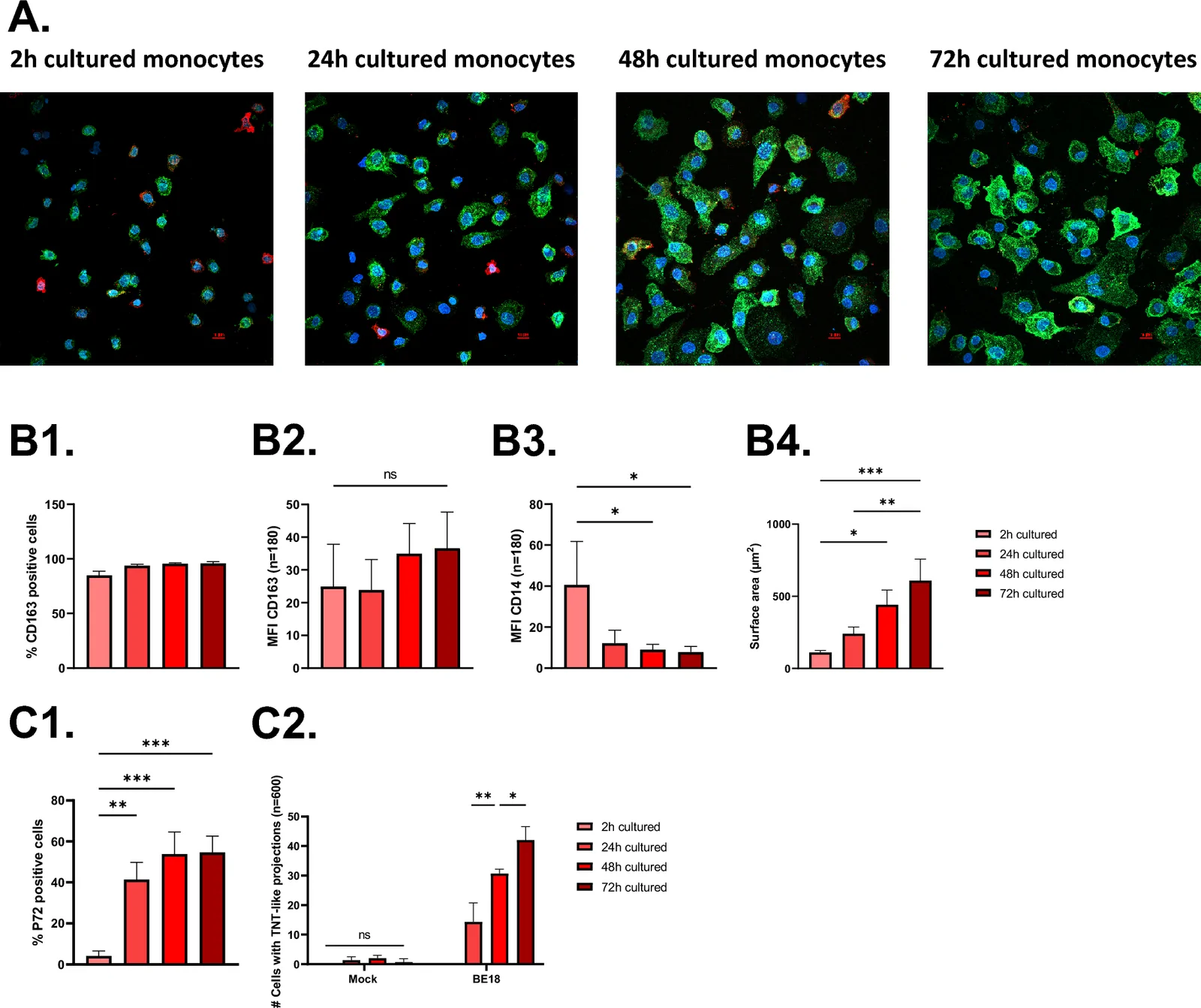
**Comparison of ASFV-susceptibility and TNT-induction of monocytes during progressive maturation into MDMs.** **A** The morphology of progressively differentiating monocytes into MDMs (2, 24, 48 and 72 h of cultivation) was assessed by fluorescence microscopy. Cells were double immunolabelled for the myeloid surface markers CD14 (red) and CD163 (green), and nuclei were counterstained with Hoechst 33342 (blue). Scale bar: 10 µm. **B1** The proportion of CD163-positive cells in the isolated monocyte populations was subsequently evaluated by immunofluorescence microscopy. **B2–B3** Quantitative image analysis of confocal microscopy images was performed to determine the MFI of CD163 and CD14 **B2**, as well as the mean cellular surface area **B3**. **C1** At 18 hpi (MOI = 1), cells were fixed and fluorescently labelled to quantify p72-positive cells following visualization of nuclei, the actin cytoskeleton and the viral p72 antigen. **C2** Subsequently, 600 mock-infected and 600 p72-positive cells per condition were manually scored by fluorescence microscopy for the presence of one or more straight, F-actin-containing cellular protrusions to enable a comparative analysis. Statistical significance is indicated as ^***^p < 0.001, ^**^p < 0.01 and ^*^p < 0.05.

After 2 h of cultivation, monocytes predominantly exhibited a small, spherical morphology, with only a minority of cells showing small morphological deviations (Figure 2A). As culture duration increased, cells progressively adopted a more spread-out phenotype, accompanied by increasing morphological heterogeneity. At 24 h of cultivation, a proportion of cells retained the spherical morphology characteristic of 2 h cultured cells, whereas others displayed more extensive spreading. This variability diminished over time, and by the later time points, the majority of cells had transitioned to an extended, flattened morphology. Ultimately, most cells exhibited the characteristic “fried egg” appearance described for mature MDMs.

Unmatured cells (2 h of cultivation) showed a slightly lower fraction of CD163-positive cells (84.90% ± 3.786) compared with those maturated for longer periods (93.77% ± 1.335, 95.71% ± 0.6911 and 95.94% ± 1.669 for 24, 48 and 72 h of cultivation, respectively) (Figure 2B1). The relatively smaller, more circular nuclei observed in marker-negative cells at the earliest time point suggests a contamination by lymphocytes. In parallel, the intensity of expression of the CD163 marker tend to increase and became more homogeneous from 2 to 72 h of cultivation; however, these differences did not reach statistical significance (Figure 2B2).

In contrast, CD14 expression showed pronounced heterogeneity in unmatured cells (2 h cultivation), with a subset of cells exhibiting markedly elevated CD14 levels (MFI: 40.57 ± 21.26; Figure 2A, B3). With increasing culture time, the proportion of highly CD14-expressing cells and overall intensity of this marker progressively decreased, resulting in reduced mean CD14 expression at 24, 48 and 72 h of cultivation (12.19 ± 6.285, 8.96 ± 2.642 and 7.846 ± 2.711, respectively). Consequently, CD14 expression at 2 h cultivation differed significantly (*p* < 0.05) from that at 48 h and 72 h of cultivation.

Unlike the dynamic regulation of CD14, the mean cell surface area increased linearly with culture duration (Figure 2B4). Accordingly, 2-h-cultured monocytes (111.1 µm^2^ ± 14.56) were significantly smaller than 48-h-cultured (442.4 µm^2^ ± 101.2; *p* < 0.05) and 72-h-cultured monocytes (609.1 µm^2^ ± 149.1; *p* < 0.001). Additionally, 24-h-cultivated monocytes (241.3 µm^2^ ± 47.01) were significantly smaller than 72-h-cultivated monocytes (*p* < 0.01).

Furthermore, we observed that monocyte susceptibility to ASFV infection increased with maturation time (Figure 2C1). Unmatured macrophages (2 h cultivation) were the least susceptible, with only 4.18% ± 2.406 staining positive for ASFV p72, which is comparable with the data obtained in PAMs earlier. After 24 h of cultivation, infection rates rose sharply to 41.34% ± 8.436 (*p* < 0.01 compared with 2-h-cultivated monocytes). A further, though less pronounced, increase was observed at 48 h cultivation (53.83% ± 10.81; *p* < 0.001 compared with 2-h-cultivated monocytes). Susceptibility remained stable thereafter, with 72-h-cultivated monocytes showing comparable infection levels with 48-h-cultivated monocytes (54.54% ± 8.005).

This temporal pattern differed from the kinetics of TNT-like projection formation among p72-positive cells (Figure 2C2). Interestingly, no TNT-like structures were detected in infected 2-h-cultivated monocytes. The 24 h-cultivated monocytes mark the first population of infected cells exhibiting projections, with 14.33 p72-positive cells out of 600 infected cells (± 6.429), indicating that the capacity to form protrusions emerged between 2 and 24 h of monocyte cultivation. This number further increased at 48 h of cultivation (30.67 ± 1.528 out of 600 infected cells), representing a significant rise relative to 24 h of cultivation (*p* < 0.01). The highest number of projection-bearing infected cells was observed in 72-h-cultivated MDMs (42.00 ± 4.583 out of 600 infected cells), significantly exceeding the 48-h-cultivation level (*p* < 0.05).

Overall, all four maturation stages were susceptible to ASFV infection, with susceptibilities increasing up to 48 h of cultivation and stabilizing thereafter. Beginning at 24 h of cultivation, ASFV-infected monocytes acquired the ability to form TNT-like projections, and the prevalence of such structures increased progressively with further maturation.

## Polarized macrophages display reduced quantities of TNT-like projections

Based on previously reported differences in the susceptibility of polarized macrophages to ASFV infection, we investigated whether these macrophage subsets also differ in their capacity to induce TNT-like projections following ASFV infection. To address this question, monocyte-derived macrophages (MDMs) were differentiated into MDM1 and MDM2. Successful differentiation into three distinct experimental groups was confirmed by immunolabelling for CD163 and MHC-II, as well as measurement of the mean cell surface area of the populations. The expression levels of these markers, together with cellular surface area, were quantified by confocal immunofluorescence microscopy. As the cells originated from the same animals used in the monocyte maturation experiments, an additional assessment of the percentage of CD-163 positive cells was deemed less relevant.

**Figure 3:**
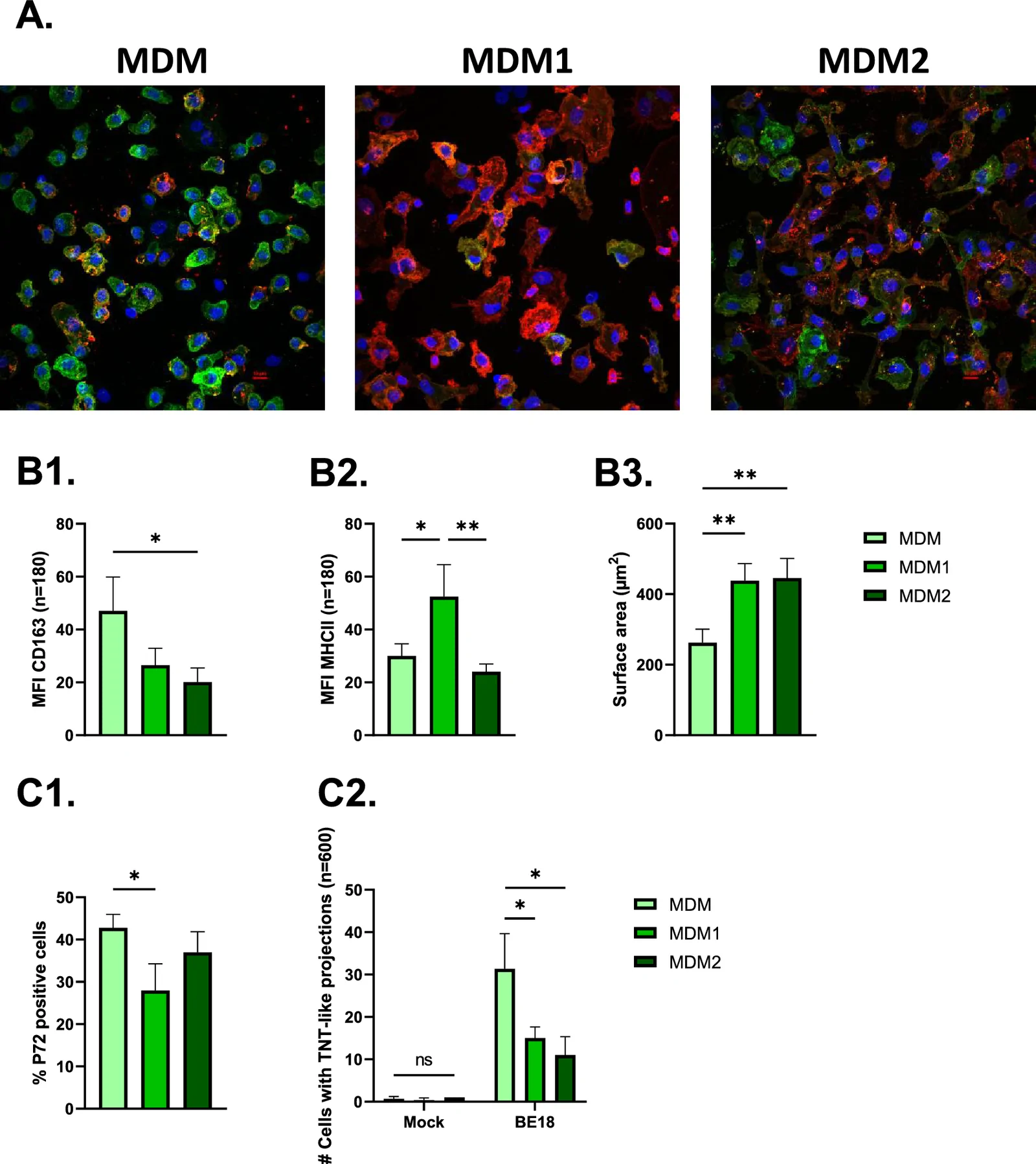
**Comparison of ASFV susceptibility and TNT induction of non-polarized and polarized MDMs.** **A** The morphology of non-polarized (MDM) and polarized MDMs (MDM1 and MDM2) was assessed by fluorescence microscopy. Cells were double-stained for CD163 (green) and MHC-II (red), and nuclei were counterstained with Hoechst 33342 (blue). Scale bar: 10 µm. **B1–B3** Quantitative image analysis of confocal microscopy images was performed to determine the MFI of CD163 **B1** and MHC-II **B2**, as well as the mean cellular surface area **B3**. **C1** At 18 hpi (BE18, MOI = 1), cells were fixed and fluorescently labelled to quantify p72-positive cells following visualization of nuclei, the actin cytoskeleton and the viral p72 antigen. **C2** Subsequently, 600 mock-infected and 600 p72-positive cells per condition were manually scored by fluorescence microscopy for the presence of one or more straight, F-actin-containing cellular protrusions to enable a comparative analysis. Statistical significance is indicated as ^**^p < 0.01 and ^*^p < 0.05.

Compared with undifferentiated MDMs, both MDM1 and MDM2 displayed a more irregular and extensively spread surface morphology (Figure 3A). Of note, light microscopical observations (data not shown) revealed that MDM1 tended to aggregate, forming clustered groups of macrophages, in contrast to MDMs and MDM2. MDM2 cells, in turn, exhibited a more elongated morphology than both other groups, which presented a more spherical morphology (Figure 3A).

With regard to surface marker expression, undifferentiated MDMs showed a higher MFI of CD163 (47.02 ± 12.88) compared with MDM1 (26.48 ± 6.432) and MDM2 (20.10 ± 5.329) (Figure 3B1). Specifically, CD163 expression was significantly higher in MDMs than in MDM2 (*p* < 0.05).

Conversely, MDM1 exhibited the highest expression of MHC-II (52.44 ± 12.13) compared with both MDMs (29.97 ± 4.606) and MDM2 (24.02 ± 2.937) (Figure 3B2), resulting in statistically significant differences between MDM1 and MDMs (*p* < 0.05) and between MDM1 and MDM2 (*p* < 0.01).

Despite these differences in surface antigen expression, MDM1 and MDM2 displayed comparable cellular surface areas (438.5 µm^2^ ± 48.04 and 445.6 µm^2^ ± 55.42, respectively), both of which were significantly larger than that of undifferentiated MDMs (262.2 µm^2^ ± 38.42; *p* < 0.01) (Figure 3B3).

Undifferentiated MDMs exhibited a higher number of p72-positive cells at 18 hpi (42.77% ± 3.227) compared with MDM1 (27.93% ± 6.324) and MDM2 (36.97% ± 4.891), resulting in a statistically significant difference between MDMs and MDM1 (*p* < 0.05) (Figure 3C1).

Notably, a statistically significant difference in the number of TNT-like projections was also observed between MDMs and both polarized macrophage populations (*p* < 0.05). Specifically, MDMs displayed approximately twice as many p72-positive cells (31.33 ± 8.327) with protrusions as either MDM1 (15 ± 2.646) or MDM2 (11 ± 4.359) out of 600 ASFV-infected cells (Figure 3C2). This is in contrast with the number of cells with protrusions in the mock-infected MDMs, MDM1 and MDM2, which all presented negligible numbers of cells with projections (0.57 ± 0.58, 0.33 ± 0.58 and 1.00 ± 0.00, respectively).

## Induction of TNT-like projections is conserved across ASFV strains despite pronounced strain-specific differences

**Figure 4:**
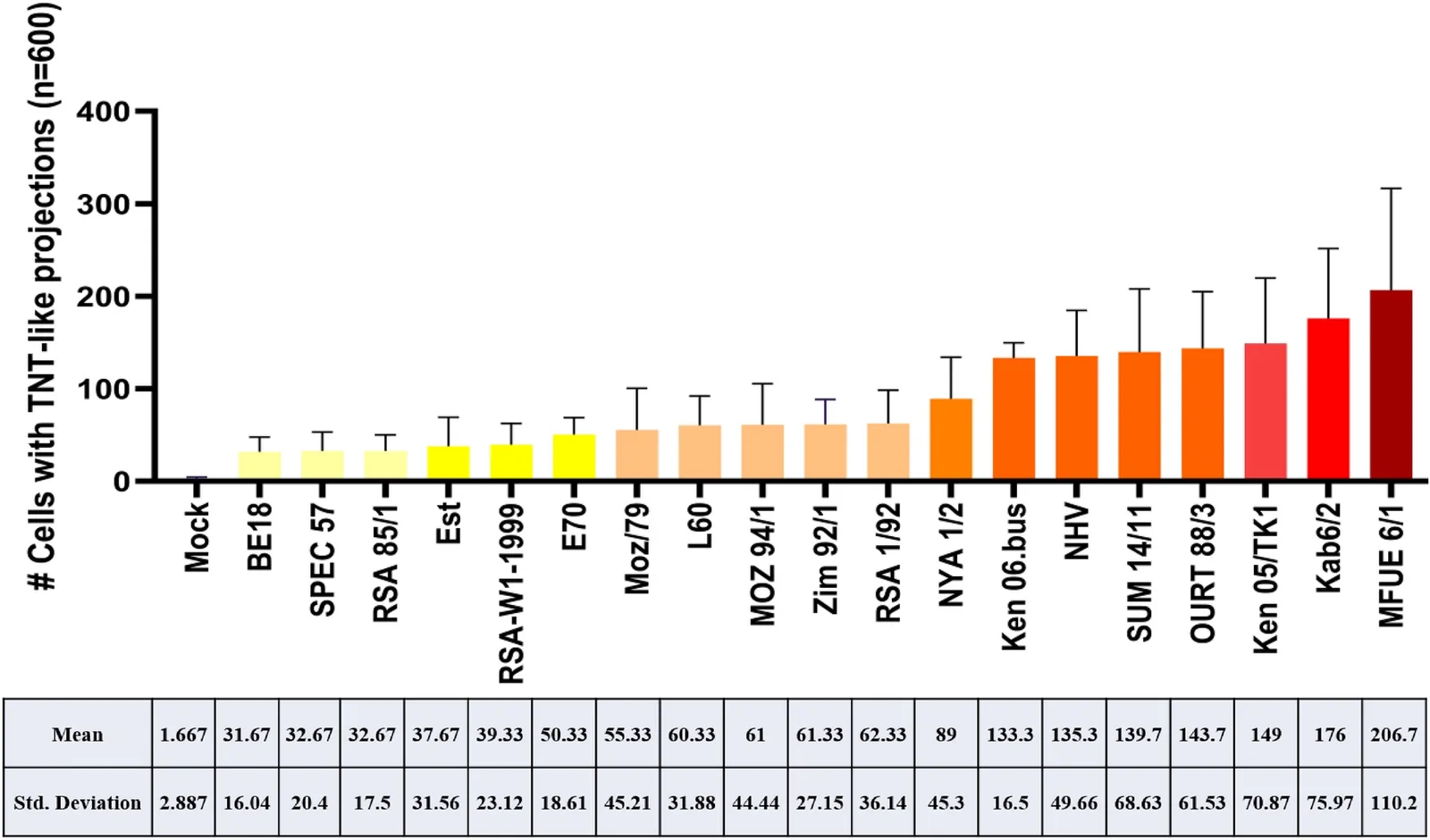
**Comparison of different ASFV isolates on the capacity to induce TNT-like projections.** A screening assay was conducted using 19 ASFV isolates representing 14 genotypes and three virulence profiles to assess their capacity to induce TNT-like projections upon infection (MOI = 0.1). At 24 hpi, cells were fixed and fluorescently labelled to visualize the nuclei, actin cytoskeleton and the viral p72 antigen. Subsequently, 600 mock-infected and 600 p72-positive cells per strain were manually scored by microscopy for the presence of one or more straight, F-actin-containing cellular protrusions to enable a comparative analysis. A high degree of variation, independent of genotype or virulence, can be observed.

To assess whether the ability of ASFV to induce TNT-like projections in fully maturated (96 h of culture) MDMs is conserved among different virus isolates, a panel of 19 selected field isolates representing multiple genotypes or virulence profiles was analysed. In addition, the Vero cell-adapted strain Ba71V was included to evaluate TNT-like projection induction in a continuous cell line which is commonly used to study ASFV–host cell interactions. MDMs were infected with the different strains at a MOI of 0.1, and the proportion of cells exhibiting TNT-like projections was quantified at 24 hpi by analysing 600 cells per condition, including 600 infected cells for each infected condition and 600 randomly selected cells for the mock-infected condition (Figure 4).

All tested strains were able to induce TNT-like projections in infected MDMs (Figure 4). However, a high degree of variability was observed between the isolates. Surprisingly, infection with BE18, used to describe ASFV-induced TNT-like projections during a previous study [23], induced the lowest proportion of cells with protrusions, with 31.67 cells out of 600 p72-positive cells (± 16.04). This is in contrast with MFUE6/1-infected MDMs, which resulted in the most cells with projections in this screening assay (206.7 ± 110.2).

**Figure 5:**
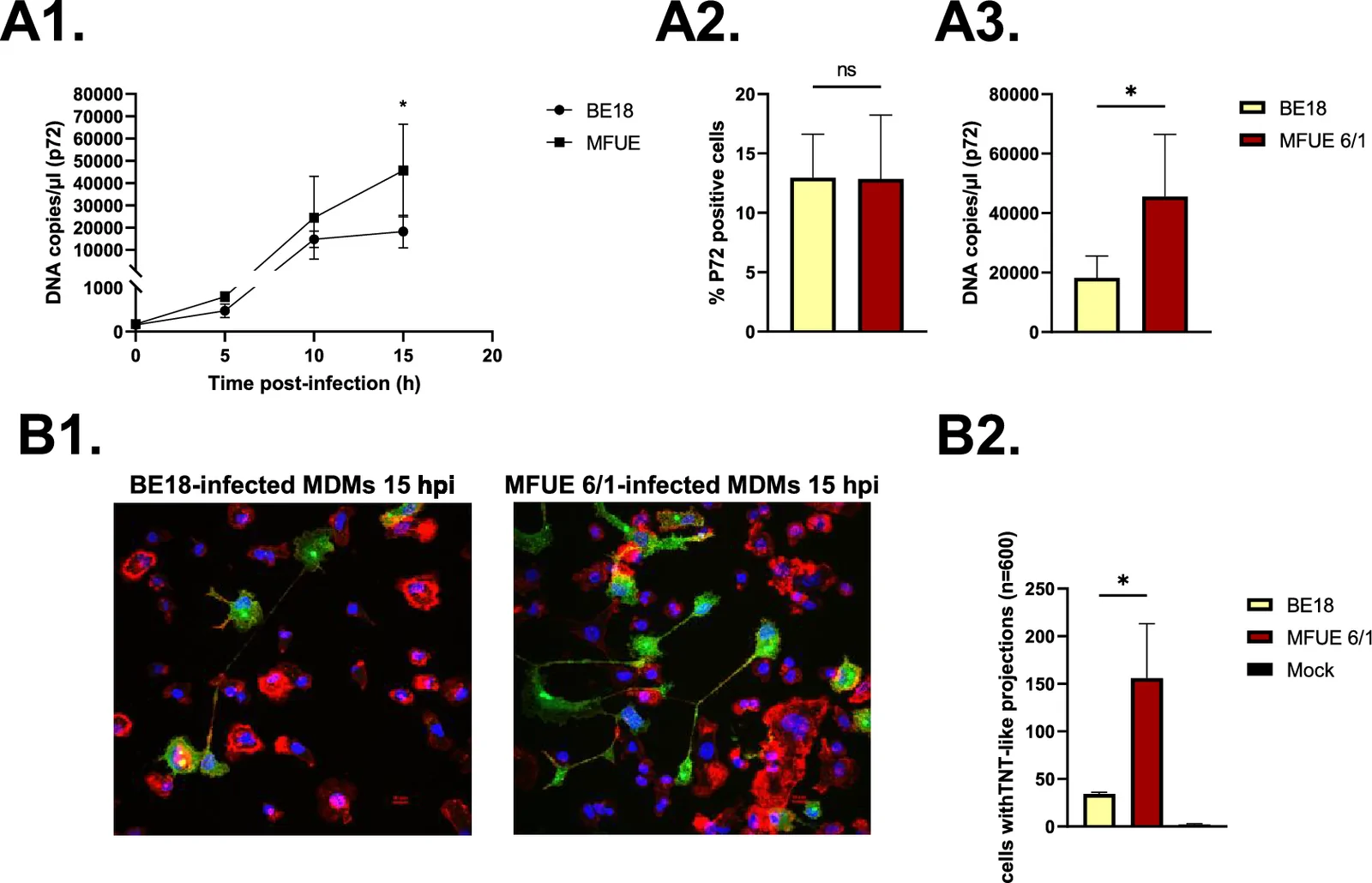
**Comparative one-step growth kinetics and quantification of cells with TNT-like projections in ASFV BE18 and MFUE6/1 infected MDMs.** **A1** Monocyte-derived macrophages (MDMs) were synchronously infected with ASFV strains BE18 or MFUE 6/1 (MOI = 1). Cell culture supernatants were collected at 0, 5, 10 and 15 hpi and extracellular viral DNA was quantified by real-time qPCR. **A2** At 15 hpi, cells were fixed with 3% paraformaldehyde (PFA), and fluorescently labelled to visualize the nuclei, actin cytoskeleton and the viral p72 antigen. No significant difference in the number of infected cells was observed at 15 hpi. **A3** At 15 hpi, MFUE 6/1-infected MDMs released significantly higher numbers of ASFV genome copies into the supernatant (45 600 ± 20 819.78 copies/µL) than BE18-infected MDMs (18 243.33 ± 7355.19 copies/µL). **B1** Representative confocal micrographs of ASFV-infected MDMs (yellow arrows) exhibiting TNT-like projections. Scale bar = 10 µm. Cells infected with MFUE 6/1 frequently displayed more highly branched protrusions (yellow arrowheads) than BE18-infected cells. **B2** To compare protrusion formation, 600 mock-infected cells and 600 p72-positive cells per virus strain were manually scored for the presence of one or more straight, F-actin-positive cellular protrusions. Statistical significance is indicated as ^*^ p < 0.05.

Because the experimental set-up does not result in a uniform or synchronous infection, subtle differences may become amplified at the selected time point (24 hpi) [53]. Therefore, to further investigate the observed variation between the strains inducing the lowest and the highest number of cells with TNT projections (BE18 and MFUE 6/1, respectively), their replication kinetics were compared using a one-step growth kinetics assay (Figure 5).

Immediately after removal of the inoculum (0 hpi), comparable ASFV DNA copy numbers were detected in the supernatants of MFUE 6/1- and BE18-infected MDMs (174.64 ± 39.71 and 156.68 ± 47.60 copies/µL, respectively). At 5 and 10 hpi, MFUE 6/1 consistently yielded higher extracellular viral DNA copy numbers, although not significantly different than BE18 (at 5 hpi, 796.66 ± 111.29 versus 473.11 ± 155.80 copies/µL; at 10 hpi, 24 467.78 ± 18 585.23 versus 14,731.11 ± 3651.74 copies/µL) (Figure 5A1). In contrast, at 15 hpi, supernatants from MFUE 6/1-infected MDMs contained significantly higher ASFV DNA copy numbers than those from BE18-infected cells (45 600 ± 20 819.78 versus 18 243.33 ± 7355.19 copies/µL; *p* < 0.05), despite a comparable number of infected cells (Figure 5A2 and A3).

Consistent with the higher extracellular viral DNA levels observed at 15 hpi, MFUE 6/1 infection resulted in a significantly higher number of infected MDMs exhibiting TNT-like projections than BE18 infection (respectively, 156.00 ± 57.30 versus 34.33 ± 1.53 cells displaying TNT-like projections out of 600 infected cells analysed per condition; *p* < 0.05) (Figure 5B2).

**Figure 6:**
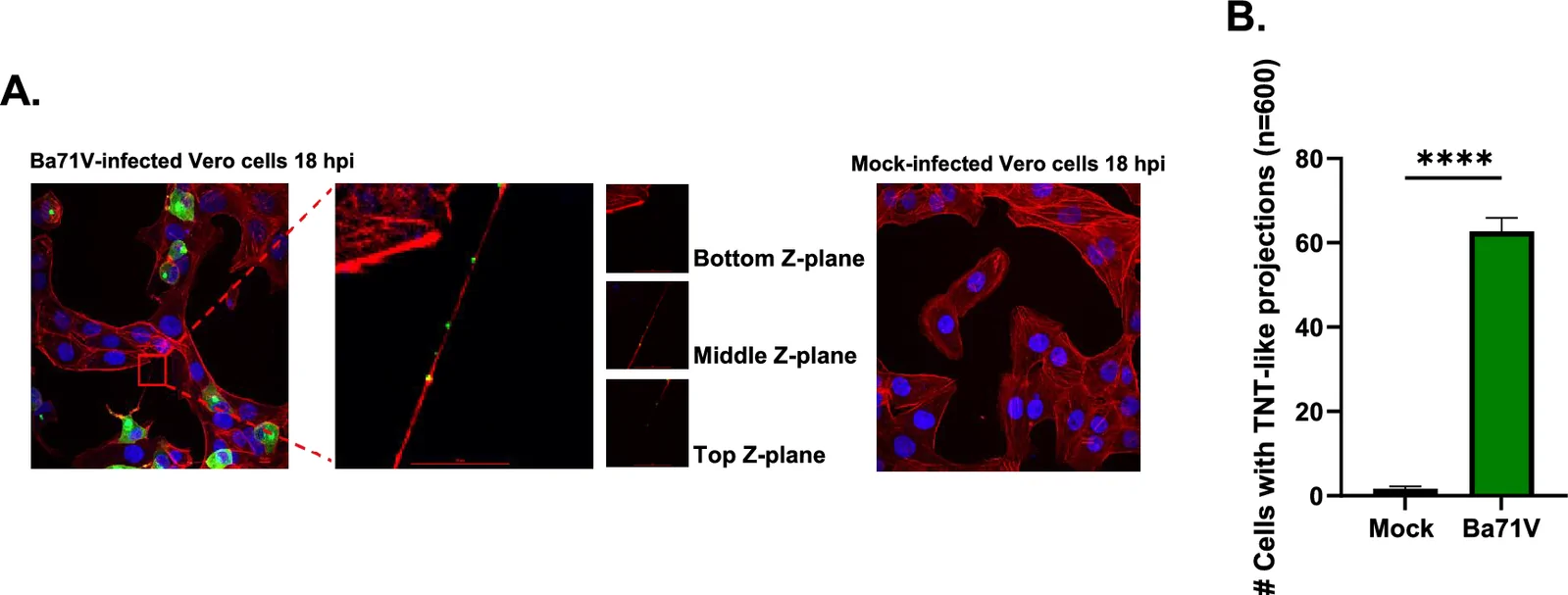
**ASFV-infection of Vero cells leads to enhanced formation of TNT-like projections.** **A** Representative images of ASFV- and mock-infected Vero cells 18 hpi (MOI = 10). The cells were stained for the presence of viral p72 (green) with a mouse monoclonal antibody (1BC11), F-actin (red) with Phalloidin-iFluor 594 and DNA with Hoechst 33342. Long, straight F-actin protrusions were observed in a fraction of the p72-positive cells. A close-up view of an ASFV-infected Vero cell reveals a TNT-like projection containing P72-positive foci, extending towards the cell body of a distant cell making contact at the apical side (middle and top *z*-plane). Scale bar: 10 μm. **B** Furthermore, at this time point, 600 mock-infected and 600 p72-positive cells were manually scored by microscopy for the presence of one or more straight, F-actin-containing cellular protrusions to enable a comparative analysis. Statistical significance is indicated as ^****^p < 0.0001.

Additionally, similar long, straight F-actin-containing protrusions that did not contact the substrate on which the cells are seeded and allowed contact with distant cells were observed in Ba71V-infected Vero cells (Figure 6A), with significantly more p72-positive Vero cells displaying protrusions, 62.67 cells out of 600 p72-positive cells (± 3.22), compared with mock-infected Vero cells (1.67 ± 0.58) at 18 hpi (*p* < 0.0001) (Figure 6B).

## Discussion

In this study, we compared the ability of distinct porcine macrophage populations and Vero cells to support ASFV-induced TNT-like projection formation and assessed whether this phenotype differs among ASFV strains of diverse genotypes and virulence.

We first investigated whether macrophages isolated from different organs differ in their capacity to generate TNT-like projections following ASFV infection. Based on our previous observation that PAMs exhibit reduced ASFV cell-to-cell spread compared with MDMs and splenic macrophages [20], we hypothesized that tissue-specific macrophage characteristics influence virus-induced protrusion formation.

Our results support this hypothesis by demonstrating marked differences in TNT induction among macrophage populations. PAMs exhibited the lowest capacity to form TNT-like projections following ASFV infection. Splenic macrophages also displayed a reduced capacity for TNT induction when normalized to the number of infected cells, whereas MDMs showed the highest propensity to generate TNT-like projections, with this capacity varying according to their maturation stage and polarization state.

Before comparing TNT formation, we confirmed by immunofluorescence microscopy that the three macrophage populations differed in their susceptibility to ASFV infection. These observations were consistent with cellular marker and infection profiles previously reported by flow cytometry for PAMs and for MDMs at different stages of differentiation [54] and polarization [19]. Comparable flow cytometry data are not currently available for splenic macrophages. The lower susceptibility observed in splenic macrophage cultures may partly reflect the heterogeneous nature of the isolation procedure, which can yield a mixture of monocytes, dendritic cells and multiple splenic macrophage subsets [8, 55]. Indeed, a small population of rounded cells resembling immature MDMs was observed in these cultures. Since monocytes are less susceptible to ASFV infection than differentiated macrophages [54], their presence likely contributed to the lower proportion of infected cells compared with MDMs, although this difference did not reach statistical significance. This observation is consistent with previous findings [20]. A more detailed characterization linking specific splenic myeloid populations to ASFV susceptibility and TNT formation would provide valuable insights but was beyond the scope of the present study.

Differences in macrophage maturation status further influenced TNT induction. Luminal myeloid cells, represented by monocytes and PAMs, exhibited a smaller and more rounded morphology than MDMs and splenic macrophages. During monocyte differentiation into MDMs, cells progressively spread and acquired a more heterogeneous morphology, a feature even more pronounced in splenic macrophages. Interestingly, TNT-like projections following BE18 infection were only observed in more mature macrophage populations, with the capacity to generate these structures emerging between 2 and 24 h of differentiation. This suggests that macrophage maturation is accompanied by cellular changes enabling protrusion formation.

Polarization further modulated this phenotype. MDM2 displayed a more elongated morphology than MDM1, and MDM1 contained significantly fewer infected cells exhibiting TNT-like projections than non-polarized MDMs. Although MDM1 consistently generated fewer protrusions than MDM2, this difference did not reach statistical significance. This reduced capacity is unlikely to be explained solely by differences in cell density, as lower intercellular distances did not increase TNT formation (Additional file 1). Instead, polarization-associated changes in cellular functions, including actin dynamics and viral entry pathways, may contribute to this phenotype. In particular, the lower infection rate observed in MDM1 may be associated with reduced Rac activation, which is required for macropinocytosis, one of the ASFV entry mechanisms [56, 57].

The marked morphological differences observed among macrophage populations suggest that variations in cytoskeletal organization may contribute to their differential capacity to generate TNT-like projections. The actin cytoskeleton plays a central role in maintaining cell morphology and driving membrane remodelling, while also forming the structural backbone of TNTs [58, 59]. Although comparative studies of actin regulation across primary macrophage populations remain limited, TNT biogenesis is known to depend on actin remodelling mediated by Rho-family GTPases, including Rho, Rac1 and Cdc42, which regulate cell shape, polarity and migration [60–62].

Increased expression of Rho and Cdc42 during macrophage differentiation has been reported in murine models [63] and may similarly contribute to the increased ability of porcine MDMs to generate TNT-like projections. Conversely, polarization-associated changes in actin regulation may modify the availability or activity of cellular factors required for ASFV-induced protrusion formation.

To facilitate future mechanistic investigations, we evaluated Vero cells as a standardized complementary model. ASFV-infected Vero cells formed F-actin-containing membrane protrusions displaying the defining characteristics of TNTs, consistent with previous observations in this cell type [64]. The occurrence of these structures in a non-porcine cell model suggests that ASFV may exploit conserved host pathways involved in actin regulation, further supporting a role for Rho-family GTPase signalling in TNT formation.

All ASFV isolates investigated induced TNT-like projections, indicating that this phenotype is conserved across genetically diverse strains. However, the proportion of infected cells displaying protrusions varied considerably between isolates. MFUE 6/1 induced significantly more TNT-like projections than BE18 and also produced higher extracellular viral DNA levels per infected cell, suggesting that TNT induction may be associated with viral replication efficiency. Consistently, the low-virulence genotype I strains NHV and OURT88/3 induced more frequent protrusion formation than the virulent genotype I strains L60 and E70, in agreement with previous reports describing differences in ASFV replication capacity in vitro [65–67].

Since membrane protrusions can promote antibody-resistant cell-to-cell dissemination in several porcine viruses [27, 28], enhanced TNT formation may contribute to the persistence associated with some lower-virulence ASFV strains [68, 69]. Quantifying additional parameters, such as the number and length of projections per cell, will be necessary to further define their functional relevance during ASFV infection. Nevertheless, conflicting observations regarding ASFV replication kinetics [20, 70] indicate that further studies are required to clarify the relationship between replication, virulence and TNT formation.

To limit variability associated with virus stock production, all ASFV stocks were prepared under standardized conditions using MDMs derived from the same donor animal and collected at the same time point. Therefore, differences in TNT induction between strains are unlikely to result from variations in the cellular substrate used for virus propagation.

Strain-dependent differences may also result from viral genetic determinants. The close genetic relationship between NHV and L60 [35] makes these strains particularly suitable for further comparative genomic analyses aimed at identifying viral factors involved in protrusion regulation.

BE18 induced the lowest proportion of infected cells with TNT-like projections, despite being the strain in which these structures were initially described [23]. This difference cannot be attributed solely to extensive in vitro passage, as NHV displayed strong TNT induction despite a similarly higher passage history. Likewise, differences in virus stock titres are unlikely to explain the observed variation, as strains with comparable titres (e.g. OURT88/3, RSA 1/92 and RSA-W1-1999) displayed distinct capacities to induce protrusions. Finally, soluble mediators released during infection are unlikely to be sufficient drivers of TNT formation. Conditioned medium from BE18- or MFUE 6/1-infected macrophages neither induced TNT-like projections in mock-infected cells nor modified TNT formation in BE18-infected cells (Additional file 2), indicating that productive infection is required. Although ASFV strains differentially modulate cytokine responses [71, 72], our present results do not support a mechanism analogous to the cytokine-mediated induction of TNTs described for human immunodeficiency virus type 1 (HIV-1) [73].

In conclusion, macrophage tissue origin, maturation stage and polarization state strongly influence the capacity to generate ASFV-induced TNT-like projections. These differences likely reflect variations in cellular differentiation and cytoskeletal regulation. Vero cells provide a practical complementary model for mechanistic studies, while strain-dependent differences indicate that both viral and host factors regulate TNT induction. Together, these findings provide new insights into ASFV-induced membrane remodelling and establish a basis for future studies investigating the contribution of TNT-like structures to viral dissemination and pathogenesis.

## Supplementary information

**Additional file 1**. **Induction of TNT-like projections is independent of seeding density.**

**Additional file 2. Induction of TNT-like projections across ASFV strains is independent of soluble mediators.**

## Data Availability

The data that support the findings of this study are available on reasonable request from the authors.
