# Supplementary material for: Strain and cell type dependent induction of tunnelling nanotube-like projections by African swine fever virus
Source: Vet Res. 2026 Oct 30;57:191. doi: 10.1186/s13567-026-01866-z (PMC13721319; doi:10.1186/s13567-026-01866-z)
Supplement: Supplementary file 1 [file 13567_2026_1866_MOESM1_ESM.docx]

## **Additional file 1.**

## **Induction of TNT-Like Projections is Independent of Seeding Density**

***Materials and methods***

Monocytes were seeded at different densities (3x10^5^, 4x10^5^ and 5x10^5^ monocytes/well respectively) in 48-well plates on coverslips and left to maturate towards MDMs like described in the materials and methods section. After this, the three different conditions were infected with BE18 (MOI=1). At 18 hpi, the cells were fixed and fluorescently labelled to visualize the nuclei, actin cytoskeleton, and the viral p72 antigen. 600 mock-infected and 600 p72-positive cells per condition were manually scored by microscopy for the presence of one or more straight, F-actin-containing cellular protrusions.

***Results***

To determine whether intercellular distance has an effect on the induction of TNT-like projections after ASFV-infection (BE18, MOI=1), monocytes were seeded at different densities (3x10^5^, 4x10^5^ and 5x10^5^ monocytes/well respectively) in 48-well plates on coverslips and left to maturate towards MDMs. At 18 hpi, similar numbers of ASFV-infected cells with TNT-like projections were observed between all three conditions (Additional Figure 1).

**Additional Figure 1. Effect of seeding density on the formation of ASFV-induced TNT-like projections.**
Monocytes were seeded at different densities in 48-well plates on coverslips and left to maturate towards MDMs. After this, the three different conditions were infected with BE18 (MOI=1). At 18 hpi, the cells were fixed and fluorescently labelled to visualize the nuclei, actin cytoskeleton, and the viral p72 antigen. 600 mock-infected and 600 p72-positive cells per condition were manually scored by microscopy for the presence of one or more straight, F-actin-containing cellular protrusions. No statistically significant difference could be observed between all three ASFV-infected groups.
