# Supplementary material for: Strain and cell type dependent induction of tunnelling nanotube-like projections by African swine fever virus
Source: Vet Res. 2026 Oct 30;57:191. doi: 10.1186/s13567-026-01866-z (PMC13721319; doi:10.1186/s13567-026-01866-z)
Supplement: Supplementary file 2 [file 13567_2026_1866_MOESM2_ESM.docx]

## **Additional file 2.**

## **Induction of TNT-Like Projections Across ASFV Strains is Independent of Soluble Mediators**

***Materials and methods***

To investigate the contribution of infection-induced soluble mediators, MDMs (1 × 10⁶ cells per well) seeded in 24-well plates containing 13 mm No. 1 coverslips (Epredia) were infected separately with ASFV isolates BE18 and MFUE6/1 with a MOI of 0.1, or mock-infected, according to the previously described infection protocol. At 24 hpi, culture supernatants were collected and passed through a 0.1 µm low–protein-binding syringe filter (Merck) to remove infectious viral particles.

Subsequently, MDMs (5 × 10⁵ cells per well) seeded in 48-well plates containing coverslips were infected with BE18 (MOI = 0.1) or incubated with RPMI alone as a mock-infected control, following the same infection protocol. Immediately after infection, the cells were washed 2 times with Hanks’ balanced salt solution (HBSS) (Gibco) and subsequently incubated with the filtered supernatants derived from the three differentially conditioned media. After 24h of incubation, cells were fixed and analyzed for the presence of TNT-like projections by immunofluorescence staining.

***Results***

To investigate whether the observed differences in the abundance of cells bearing TNT-like projections were attributable to strain-specific induction of soluble mediators following infection, supernatants were collected from MDMs infected with either a high-inducing strain (MFUE 6/1) or a low-inducing strain (BE18). The supernatants were filtered through a 0.1 µm low–protein-binding membrane to remove infectious virus and subsequently applied to BE18-infected or mock-infected MDMs (Additional Figure 2).

**Additional Figure 2. Effect of soluble mediators on the formation of ASFV-induced TNT-like projections.**
(**A**) MDMs were infected with medium alone (Mock), BE18 or MFUE 6/1 (MOI=0.1). Twenty-four hpi, the supernatants were collected and the cells were fixed and fluorescently labelled to visualize the nuclei, actin cytoskeleton, and the viral p72 antigen. 600 mock-infected and 600 p72-positive cells per strain were manually scored by microscopy for the presence of one or more straight, F-actin-containing cellular protrusions. (**B**) The collected supernatants were cleared from infectious virions and incubated with MDMs infected with medium alone (Mock) or BE18 (MOI=0.1). After 24 hours incubation, cells were fixed, stained and analysed as described. Statistical significance is indicated as **p < 0.01.

The absence of infectious virus in the filtered supernatants was confirmed by the lack of P72-positive cells, as determined by immunofluorescence microscopy, in mock-infected MDMs 24 hours after incubation with supernatants derived from BE18- or MFUE 6/1-infected cultures. This finding was further supported by negative virus titration results obtained from supernatants collected 24 hours after their application to mock-infected cells.

Consistent with the initial screening and confirmatory assays of synchronously infected cells at 15 hpi, marked differences in the number of TNT-like projections were observed between BE18- and MFUE 6/1-infected cells that produced the conditioned media (Additional Figure 2A). Significantly more MFUE 6/1-infected cells exhibited TNT-like projections (104.7 ± 26.27) compared with BE18-infected cells (25.0 ± 1.00; p < 0.01).

In contrast, BE18-infected cells incubated with BE18-, MFUE 6/1-, or mock-conditioned supernatants displayed comparable numbers of cells bearing TNT-like projections (31.33 ± 6.81, 31.33 ± 6.11, and 33.0 ± 5.0, respectively) (Additional Figure 2B). These values were similar to those observed in the original BE18-infected cultures from which the conditioned supernatants were derived.
